# Supplementary material for: Stress burden related to postreperfusion syndrome may aggravate hyperglycemia with insulin resistance during living donor liver transplantation: A propensity score-matching analysis
Source: PLoS One. 2020 Dec 10;15(12):e0243873. doi: 10.1371/journal.pone.0243873 (PMC7728193; doi:10.1371/journal.pone.0243873)
Supplement: S1 Table — (DOCX) [file pone.0243873.s001.docx]

**S1 Table.** Associations of demographic factors with postreperfusion syndrome in the entire study population (n = 324)

|  | **Univariable logistic regression** | | | | **Multivariable logistic regression** | | | |
| --- | --- | --- | --- | --- | --- | --- | --- | --- |
|  | ***ß*** | **Odds ratio** | **95% CI** | ***p*** | ***ß*** | **Odds ratio** | **95% CI** | ***p*** |
| **Demographic factors** |  |  |  |  |  |  |  |  |
| Age (years) | 0.013 | 1.013 | 0.988 - 1.038 | 0.307 |  |  |  |  |
| Sex (female) | 0.26 | 1.298 | 0.784 - 2.148 | 0.311 |  |  |  |  |
| Body mass index (kg/m^2^) | -0.001 | 0.999 | 0.943 - 1.057 | 0.963 |  |  |  |  |
| Psoas muscle index (mm^2^/m^2^) | -0.001 | 0.999 | 0.998 - 1.0 | 0.008 |  |  |  |  |
| Comorbidity |  |  |  |  |  |  |  |  |
| Hypertension | -0.31 | 0.733 | 0.419 - 1.284 | 0.278 |  |  |  |  |
| Diabetes mellitus | 0.057 | 1.058 | 0.639 - 1.752 | 0.826 |  |  |  |  |
| MELD score (points) | 0.045 | 1.046 | 1.023 - 1.069 | <0.001 | 0.033 | 1.034 | 1.008 - 1.059 | 0.009 |
| Hepatocellular carcinoma | -0.713 | 0.49 | 0.303 - 0.794 | 0.004 |  |  |  |  |
| Hepatic complications |  |  |  |  |  |  |  |  |
| Encephalopathy  (West-Haven criteria I or II) | 0.332 | 1.394 | 0.881 - 2.207 | 0.156 |  |  |  |  |
| Varix | 0.088 | 1.092 | 0.652 - 1.829 | 0.737 |  |  |  |  |
| Ascites ≥ 1L | 1.011 | 2.748 | 1.702 - 4.435 | <0.001 | 0.712 | 2.039 | 1.196 - 3.474 | 0.009 |
| Transthoracic echocardiography |  |  |  |  |  |  |  |  |
| Ejection fraction (%) | 0.024 | 1.024 | 0.969 - 1.082 | 0.394 |  |  |  |  |
| Diastolic dysfunction (≥grade II) | -1.454 | 0.234 | 0.08 - 0.683 | 0.008 | -1.541 | 0.214 | 0.071 - 0.643 | 0.006 |
| Laboratory variables |  |  |  |  |  |  |  |  |
| Hematocrit (%) | -0.061 | 0.941 | 0.909 - 0.975 | 0.001 |  |  |  |  |
| White blood cell count (x 10^9^/L) | 0.012 | 1.012 | 0.979 - 1.047 | 0.484 |  |  |  |  |
| Neutrophil (%) | 0.023 | 1.023 | 1.007 - 1.04 | 0.005 |  |  |  |  |
| Lymphocyte (%) | -0.035 | 0.965 | 0.943 - 0.988 | 0.003 |  |  |  |  |
| Albumin (g/dL) | -0.87 | 0.419 | 0.279 - 0.629 | <0.001 |  |  |  |  |
| Aspartate aminotransferase (IU/L) | 0.0 | 1.0 | 0.999 - 1.0 | 0.358 |  |  |  |  |
| Alanine aminotransferase (IU/L) | 0.0 | 1.0 | 0.999 - 1.0 | 0.236 |  |  |  |  |
| Total bilirubin (mg/dL) | 0.032 | 1.033 | 1.013 - 1.053 | 0.001 |  |  |  |  |
| Sodium (mEq/L) | -0.017 | 0.984 | 0.956 - 1.012 | 0.246 |  |  |  |  |
| Calcium (mg/dL) | -0.018 | 0.982 | 0.779 - 1.238 | 0.878 |  |  |  |  |
| Potassium (mEq/L) | -0.132 | 0.876 | 0.604 - 1.272 | 0.488 |  |  |  |  |
| Creatinine (mg/dL) | 0.192 | 1.212 | 0.995 - 1.476 | 0.056 |  |  |  |  |
| Glucose (mg/dL) | 0.002 | 1.002 | 0.998 - 1.005 | 0.385 |  |  |  |  |
| Platelet count (x 10^9^/L) | -0.005 | 0.995 | 0.99 - 0.999 | 0.018 |  |  |  |  |
| International normalized ratio | 0.062 | 1.063 | 0.959 - 1.18 | 0.245 |  |  |  |  |
| Fibrinogen (mg/dL) | -0.002 | 0.998 | 0.996 - 1.001 | 0.247 |  |  |  |  |

**Abbreviations:** CI, confidence interval; MELD, modle for end-stage liver disease
