# Supplementary material for: Stress burden related to postreperfusion syndrome may aggravate hyperglycemia with insulin resistance during living donor liver transplantation: A propensity score-matching analysis
Source: PLoS One. 2020 Dec 10;15(12):e0243873. doi: 10.1371/journal.pone.0243873 (PMC7728193; doi:10.1371/journal.pone.0243873)
Supplement: S2 Table — (DOCX) [file pone.0243873.s002.docx]

**S2 Table.** Comparison of diastolic dysfunction before surgery between all patients with/without PRS (n = 324)

| **Group** | **non-PRS** | **PRS** | ***p*** |
| --- | --- | --- | --- |
| **n** | **212** | **112** |  |
| **Diastolic dysfunction** |  |  | <0.001 |
| Normal to grade I | 183 (86.3%) | 108 (96.4%) |  |
| Grade II | 29 (13.7%) | 0 (0.0%) |  |
| Grade III | 0 (0.0%) | 4 (3.6%) |  |

**NOTE:** Values are expressed as number and proportion (%).
