# Supplementary material for: Stress burden related to postreperfusion syndrome may aggravate hyperglycemia with insulin resistance during living donor liver transplantation: A propensity score-matching analysis
Source: PLoS One. 2020 Dec 10;15(12):e0243873. doi: 10.1371/journal.pone.0243873 (PMC7728193; doi:10.1371/journal.pone.0243873)
Supplement: S3 Table — (DOCX) [file pone.0243873.s003.docx]

**S3 Table.** The rates of new-onset diabetes mellitus during the follow-up period and early bacteremia during the first 4 weeks postoperatively in PS-matched patients with and without PRS

| **Group** | **non-PRS** | **PRS** | ***p*** |
| --- | --- | --- | --- |
| **n** | **97** | **97** |  |
| Diabetes mellitus | 14 (14.4%) | 24 (24.7%) | 0.07 |
| Bacteremia | 8 (8.2%) | 15 (15.5%) | 0.12 |

**Abbreviations:** PS, propensity score; PRS, postreperfusion syndrome

**NOTE:** Values are expressed as number and proportion (%).
