# Supplementary material for: Stress burden related to postreperfusion syndrome may aggravate hyperglycemia with insulin resistance during living donor liver transplantation: A propensity score-matching analysis
Source: PLoS One. 2020 Dec 10;15(12):e0243873. doi: 10.1371/journal.pone.0243873 (PMC7728193; doi:10.1371/journal.pone.0243873)
Supplement: S4 Table — (DOCX) [file pone.0243873.s004.docx]

**S4 Table.** The rates of pre-transplant DM and post-transplant new-onset DM by LDLT etiology in PS-matched patients

| **Group** | **LDLT**  **Etiology** | **Alcoholic hepatitis** | **Hepatitis A** | **Hepatitis B** | **Hepatitis C** | **Autoimmune hepatitis** | **Drug & toxic hepatitis** | **Cryptogenic hepatitis** | ***p*** |
| --- | --- | --- | --- | --- | --- | --- | --- | --- | --- |
|  | **n** |  |  |  |  |  |  |  |  |
| **Pre-transplant overt DM** | 59 (100%) | 25 (42.4%) | 1 (1.7%) | 18 (30.5%) | 4 (6.8%) | 2 (3.4%) | 0 (0.0%) | 9 (15.3%) | 0.067 |
| **Post-transplant new-onset DM** | 38 (100%) | 9 (23.7%) | 1 (2.6%) | 17 (44.7%) | 6 (15.8%) | 4 (10.5%) | 0 (0.0%) | 1 (2.6%) | 0.107 |

**Abbreviation:** DM, diabetes mellitus; LDLT, living donor liver transplantation; PS, propensity score

**NOTE:** Values are expressed as number and proportion.
